# Stachydrine protects eNOS uncoupling and ameliorates endothelial dysfunction induced by homocysteine

**DOI:** 10.1186/s10020-018-0010-0

**Published:** 2018-03-19

**Authors:** Xinya Xie, Zihui Zhang, Xinfeng Wang, Zhenyu Luo, Baochang Lai, Lei Xiao, Nanping Wang

**Affiliations:** 1 Cardiovascular Research Center, School of Basic Medical Sciences, Xi’an Jiaotong University, Xi’an, 710061 China; 2 The Advanced Institute for Medical Sciences, Dalian Medical University, Dalian, 116044 China

**Keywords:** Stachydrine, GTPCH1, DHFR, eNOS uncoupling, Vasorelaxation

## Abstract

**Background:**

Hyperhomocysteinemia (HHcy) is an independent risk factor for cardiovascular diseases (CVDs). Stachydrine (STA) is an active component in Chinese motherwort *Leonurus heterophyllus* sweet, which has been widely used for gynecological and cardiovascular disorders. This study is aimed to examine the effects of STA on homocysteine (Hcy)-induced endothelial dysfunction.

**Methods:**

The effects of STA on vascular relaxation in rat thoracic aortas (TA), mesenteric arteries (MA) and renal arteries (RA) were measured by using Multi Myograph System. The levels of nitric oxide (NO), tetrahydrobiopterin (BH4) and guanosine 3′, 5′ cyclic monophosphate (cGMP) were determined. Endothelial nitric oxide synthase (eNOS) dimers and monomers were assayed by using Western blotting. GTP cyclohydrolase 1 (GTPCH1) and dihydrofolate reductase (DHFR) expressions were measured by using quantitative reverse transcriptase-PCR (qRT-PCR) and Western blotting.

**Results:**

STA effectively blocked Hcy-induced impairment of endothelium-dependent vasorelaxation in rat TA, MA and RA. STA-elicited arterial relaxations were reduced by NOS inhibitor NG-nitro-L-arginine methyl ester (L-NAME) or the NO-sensitive guanylyl cyclase inhibitor 1H- [1, 2, 4] Oxadiazolo[4,3-a]quinoxalin-1-one (ODQ), but not by inducible iNOS inhibitor 1400 W nor the nonselective COX inhibitor indomethacin. Hcy caused eNOS uncoupling and decreases in NO, cGMP and BH4, which were attenuated by STA. Moreover, STA prevented decreases of GTPCH1 and DHFR levels in Hcy-treated BAECs.

**Conclusion:**

We demonstrated that STA effectively reversed the Hcy-induced endothelial dysfunction and prevented eNOS uncoupling by increasing the expression of GTPCH1 and DHFR. These results revealed a novel mechanism by which STA exerts its beneficial vascular effects.

**Electronic supplementary material:**

The online version of this article (10.1186/s10020-018-0010-0) contains supplementary material, which is available to authorized users.

## Background

Stachydrine (STA) is a major constituent of Chinese motherwort *Leonurus heterophyllus* sweet, which has been used in traditional medicine to promote blood circulation and dispel blood stasis (Yin et al., 2010). STA is also highly present in *L. japonicus*, *L. cardiaca* fruits, *Leonotis leonurus* (Kuchta et al., 2013) as well as in citrus fruits (Servillo et al., 2013). Several studies have shown that STA has protective effects on vascular endothelial cells (ECs). STA protected endothelial against the injury induced by anoxia-reoxygenation (Yin et al., 2010). STA effectively reduced lipopolysaccharide (LPS)-induced endothelial inflammatory response via the inhibition of interleukin (IL-10) and thromboxane B 2 (TXB_2_) secretion (Hu et al., 2015; Hu et al., 2012). STA inhibited the deleterious effect of high glucose on ECs and acted through the modulation of SIRT1 pathway (Servillo et al., 2013). However, little is known about STA on vascular relaxation, a common feature of endothelial function.

Hyperhomocysteinemia (HHcy) is an independent risk factor for various cardiovascular diseases (CVDs) (Karolczak et al., 2013; Baggott & Tamura, 2015). Homocysteine (Hcy) exerts its adverse effect on endothelial function by increasing oxidative stress and inhibiting the activity of endothelial nitric oxide synthase (eNOS) and decreasing nitric oxide (NO) production (Cheng et al., 2015). A critical determinant of eNOS activity is its cofactor tetrahydrobiopterin (BH4). BH4 can be formed either by a de novo biosynthetic pathway using the rate-limiting enzyme GTP-cyclohydrolase I (GTPCH1) or a salvage pathway from sepiapterin, which is dependent on dihydrofolate reductase (DHFR) (Hussein et al., 2015; Haruki et al., 2016). An inadequate level of BH4 makes eNOS no longer coupled to L-arginine oxidation (eNOS uncoupling) and results in the production of reactive oxygen species (ROS) rather than NO, thereby leading to vascular endothelial dysfunction (Takimoto et al., 2005).

In this study, we investigated the effects of STA on the Hcy-induced endothelial dysfunction and with the emphasis on its role in eNOS uncoupling and the underlying mechanism.

## Methods

### Reagents

STA was obtained from Cayman Chemical (Ann Arbor, MI, USA). Dimethyl sulfoxide (DMSO), 3-[4,5-dimethylthiazol-2-yl]-2,5-diphenyl-tetrazolium bromide (MTT), acetylcholine (ACh), indomethacin, NG-nitro-L-arginine methyl ester (L-NAME), 1H- (Yin et al., 2010; Kuchta et al., 2013; Hu et al., 2015) Oxadiazolo[4,3-a]quinoxalin-1-one (ODQ), 1400 W, Hcy, angiotensin II (Ang II), palmitic acid (PA) and rabbit polyclonal antibody to GTPCH1 were purchased from Sigma-Aldrich (St. Louis, MO, USA). The antibody against eNOS was purchased from Cell Signaling Technology (Danvers, MA, USA). Mouse monoclonal antibodies to DHFR and β-actin, HRP-conjugated anti-rabbit and anti-mouse IgG polyclonal antibodies were procured from Santa Cruz Biotechnology (Santa Cruz, CA, USA). ELISA kit for cGMP was obtained from R&D Systems Inc. (R&D Systems, MN, USA). BH4 ELISA kit was from MyBioSource Inc. (San Diego, CA, USA). Dulbecco’s modified Eagle medium (DMEM), fetal bovine serum (FBS) and 4-amino-5-methylamino-2′, 7′-difluorofluorescein (DAF-FM) diacetate were obtained from Invitrogen (Carlsbad, CA, USA).

### Animals

Male Sprague-Dawley rats (8 weeks, 250–300 g) were obtained from the Experimental Animal Center of Xi’an Jiaotong University. Rats were housed in a specific pathogen-free environment under a 12 h/12 h light/dark cycle. Rats were killed with the inhalation of carbon dioxide. After death was ensured with cervical dislocation, the vessels were collected for later analysis.

### Cell culture and treatment

Bovine aorta endothelial cells (BAECs) were prepared as previously described (Wejksza et al., 2004)) and maintained in DMEM with 10% FBS, penicillin (100 U·ml^− 1^) and streptomycin (100 U·ml^− 1^). BAECs were treated with STA (0–10 μM) for 24 h or pre-treated with STA for 12 h following treatment with Hcy (500 μM) for another 12 h. BAECs within seven passages were used.

### Determination of cell viability

Cell viability was evaluated by the MTT assay. BAECs were seeded in 96-well plates and cultured until 80% confluence. Then, cells were treated with the indicated concentrations of STA for 24 h before incubation with 5 mg·ml^− 1^ MTT at 37 °C in 5% CO_2_ atmosphere for 4 h. Next, the culture medium was removed and the formazan formed in the reaction was dissolved in 150 μl DMSO. The metabolized MTT was measured by using a spectrophotometer at 490 nm, and calculated by OD (test)/OD (control) × 100%.

### Nitric oxide (NO) assay

The levels of NO in the culture supernatants were assayed with the use of the Griess Reagent Nitrite Measurement Kit (Cell Signaling) to detect nitrite, a stable and nonvolatile breakdown product of NO. Briefly, 100 μl of supernatant was mixed with 100 μl of Griess reagent in a 96-well plate. Nitrite concentration was determined by detecting spectrophotometric absorbance at 550 nm and plotted against respective concentration in a standard curve (0–100 μM) derived from the nitrite standards. Culture media from cell-free wells was used as blank control. Intracellular NO concentrations were measured by using DAF-FM diacetate (4-Amino-5-Methylamino-2′,7′-Difluorofluorescein Diacetate). Briefly, BAECs seeded on glass coverslips and treated with different stimuli. By the end of treatment, the cells were incubated with DMEM containing 5 μM DAF-FM for 30 min in the dark at 37 °C and then washed with PBS. Images were obtained using the fluorescence microscopy. NO production was evaluated by measuring fluorescence intensity.

### Measurement of BH4

BH4 levels the cell lysates were measured by using a competitive ELISA kit. Briefly, ELISA plates pre-coated with a BH4-specific monoclonal antibody were used to incubate with the samples and test standards along with fixed amount of biotin-labelled BH4. Excess sample and reagents were washed off, and avidin conjugated to HRP was added to each well and incubated again. The 3,3′,5,5’-Tetramethylbenzidine (TMB) liquid substrate was then added to each well followed by incubation for 10 min. The enzyme substrate reaction was terminated and the product was measured spectrophotometrically at 450 nm. The concentration of BH4 in each sample was then calculated by comparing the optical density to a standard curve (Almudever et al., 2013).

### Measurement of cGMP levels in rat arterial rings

Rat aortic rings were incubated at 37 °C in 12-well culture plates containing Krebs solution gassed with 95% O_2_ and 5% CO_2_. Tissue lysates were prepared by homogenization with a Polytron homogenizer and centrifugation at 12,000×g for 20 min. The levels of cGMP were measured using an enzyme immunoassay kit in accordance to the instructions of the manufacturer.

### Measurement of vasorelaxation

Rat thoracic aortas (TA), mesenteric arteries (MA) and renal arteries (RA) were harvested and immediately transferred into oxygenated ice-cold Krebs solution containing NaCl (119 mM), KCl (4.7 mM), NaHCO_3_ (25 mM), CaCl_2_ (2.5 mM), MgCl_2_ (1 mM), KH_2_PO_4_ (1.2 mM) and D-glucose (11 mM). In some experiments, the endothelial layer was denuded using 0.1% Triton X-100. After cleaned perivascular tissues, the arteries were cut into several rings (each in 2 mm length). Then the rings were suspended between two stainless steel hooks in a 5 mL organ bath filled with Krebs solution oxygenated with 95% O_2_–5% CO_2_ and maintained at 37 °C (pH 7.4) to achieve an optimal tension (TA 10 mN, RA and MA 3 mN). Each segment was allowed to equilibrate for 60 min. Curve recording was performed with LabChart™ software (AD-Instruments, Shanghai, China) by using a PowerLab Data Acquisition System™ for data acquisition. After equilibration, segments were pre-contracted with 60 mM KCl twice, and then contracted with phenylephrine (10 μM). Once a sustained tension was reached, acetylcholine (ACh, 0.01–10 μM) was added cumulatively to evoke endothelium-dependent relaxations. TAs with intact endothelia was incubated for 30 min with each of the following inhibitors: 100 μM L-NAME (an NOS inhibitor), 1 μM indomethacin (a non-selective COX inhibitor), 100 nM 1400 W (an iNOS inhibitor) and 3 μM ODQ before the contraction with phenylephrine. In organ culture experiments, aortic rings were incubated in DMEM/F12 supplemented with 10% FBS oxygenated with 95% O_2_ and 5% CO_2_ and maintained at 37 °C. After pre-incubation with 10 μM STA or vehicle for 12 h, aortic rings were mounted in Myograph system to measure the ACh-induced relaxations.

### Quantitative reverse transcriptase-PCR (qRT-PCR)

Total RNA was isolated using TRIzol (Invitrogen, Carlsbad, CA), reverse transcribed into cDNA by using iScript cDNA synthesis kit (Bio-rad, Hercules, CA). Real-time PCR was performed by using SYBR Green Supermixes (Bio-rad) and a 7500 Real-time PCR machine (Applied Biosystems, Foster City, CA). Fold changes of gene expression were calculated using the 2^-ΔΔCt^ method. The qRT-PCR primers used were as follows: GTPCH1 forward primer: 5’-TTGGAAAGGTCCATATCGGT-3′, reverse primer: 5’-ATTGTGCTCGTCACGGTTCT-3′; DHFR forward primer: 5’-AAGAACGGAAACCTGCCCTG-3′, reverse primer: 5’-GCCTCCCACTATCCAAACCA-3′; Nrf2 forward primer: 5’-AGC ACACCCAGTCAGAAACCAG-3′; reverse primer: 5’-TCTACAAACGGGAATGTCG-3′; β-actin forward primer: 5′- CGAGCATTCCCAAAGTTCTACAGTG-3′, reverse primer: 5′- CTACATACTTCCGAAAACCAGGGG-3′. β-actin was used as an internal control.

### Western blotting

Whole protein samples were extracted with lysis buffer (50 mM Tris-HCl, pH 7.5, 15 mM EGTA, 100 mM NaCl, 0.1% Triton X-100 and the protease inhibitors). Cytoplasmic protein samples were extracted with hypotonic lysis buffer (10 mM Tris-HCl, pH 7.5, 1.5 mM MgCl_2_, 10 mM KCl, and 0.5% NP40). Nuclear protein samples were extracted with high-salt buffer (20 mM Tris-HCl, 1.5 mM MgCl_2_, 420 mM NaCl, 10% glycerol, and 0.2 mM EGTA). And then separated on 10% SDS-PAGE and blotted onto PVDF membranes. The blots were incubated with primary antibodies and HRP-conjugated secondary antibodies, and then visualized by using the ECL chemoluminescence system. The intensity of the bands was quantified by using Image Pro Plus software. The eNOS dimerizations were assayed by using low-temperature SDS-PAGE (LT-PAGE) as described previously (Klatt et al., 1995). The protein lysates were mixed with loading buffer (without β-mercaptoethanol) and loaded on gels without boiling. Electrophoresis and gel transferring were kept at 4 °C during the whole procedure.

### Small interfering RNA and transfection

For small interfering RNA (siRNA)-mediated gene knockdown, the siRNA targeting the human Nrf2 gene was synthesized: sense: 5’-GCCCAUUGAUGUUUCUGAUTT-3′, antisense: 5’-AUCAGAAACAUCAAUGGGCTT-3′. The siRNAs were transfected into cells using Lipofectamine2000 (Invitrogen). A scrambled siRNA was used as negative control (NC).

### Statistical analysis

Quantitative data are expressed as mean ± SEM, and data analyses were carried out independently by the third party without knowledge of treatments. Student’s *t* test or ANOVA was used to analyze the differences between two or among more groups, respectively. Relaxation curves were analyzed by using two-way ANOVA followed by Bonferroni post-tests. Bonferroni post hoc tests were run when F achieved *P* < 0.05 and there was no significant inhomogeneity. Non-quantitative results were representative of at least five independent experiments.

## Results

### STA improved Hcy-impaired vascular relaxation

**Fig. 1:**
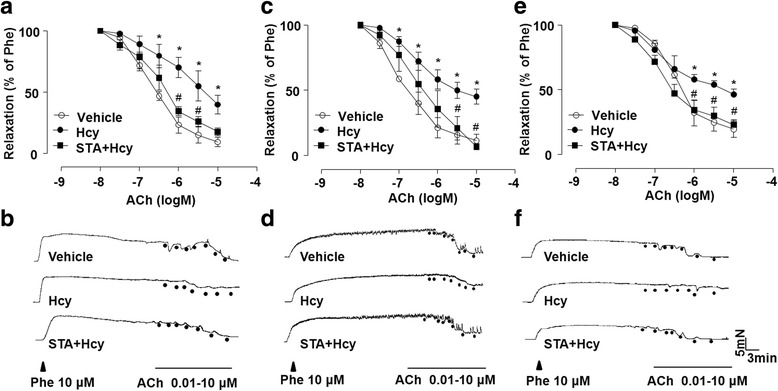
STA improved Hcy-impaired vascular relaxation. After pre-treatment of TA (**a**), MA (**c**) and RA (**e**) rings with STA (10 μM, 12 h), the arterial rings were exposed to Hcy (500 μM, 60 min). The cumulative concentration-response curves for ACh-induced relaxations of the phenylephrine-pre-contracted arterial rings were shown. **b**, **d** and **f** Representative traces of endothelium-dependent relaxations as in (**a**, **c** and **e**). Data were shown as mean ± SEM; *n* = 5 for each group, ^*^P < 0.05 vs. Vehicle; ^#^*P* < 0.05 vs. Hcy

In order to examine the effect of Hcy on vasorelaxation, rat TA, MA and RA rings were incubated with Hcy (500 μM) or control medium for 1 h before measuring the ACh-induced endothelium-dependent relaxation. The effects of STA were assessed by the pre-incubation of artery rings with STA (10 μM) for 12 h before the exposure to Hcy. As shown in Fig. 1, Hcy significantly impaired the vasorelaxations in response to ACh in all three types of arteries. Clearly, STA effectively restored the vascular relaxation to ACh. Figure 1b–f are representative traces of endothelium-dependent relaxations as in A, C and E. In uninjured rat TA, MA and RA, STA only slightly affected the vasorelaxation (Additional file 1: Figure S1A-C). Taken together, these results suggest that STA protected endothelial function impaired by Hcy.

In addition, we assessed the effects of STA on endothelial dysfunction induced by Ang II and PA, the two common injurious factors associated with metabolic disorders such as hypertension and obesity. in In rat thoracic aortas, exposure to either Ang II (1 μM, 1 h) or PA (200 μM, 1 h) significantly impaired vasorelaxations (Additional file 2: Figure S2A and B). However, pretreatment with STA (10 μM, 12 h) effectively protected the vascular relaxation against Ang II and PA.

### STA-improved relaxation was dependent on endothelium-derived NO

**Fig. 2:**
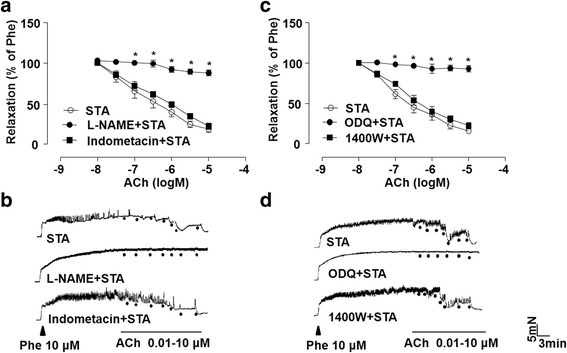
STA-improved relaxation was dependent on endothelium-derived NO. Rat thoracic artery rings were incubated with STA (10 μM) for 12 h and exposed to L-NAME (100 μM), indomethacin (1 μM) (**a**), 1400 W (100 nM) or ODQ (3 μM) (**c**) for 30 min before the PE-contraction and ACh-induced relaxation. **b** and **d** Representative traces of endothelium-dependent relaxations as in (**a** and **c**). Data were shown as mean ± SEM; *n* = 5, ^*^P < 0.05 vs. Vehicle

To define the contribution of NO in the STA-improved relaxation, the artery rings were treated with different pharmacological inhibitors before the measurement of relaxation. As shown in Fig. 2, either L-NAME (an inhibitor of NOS) or ODQ (an inhibitor of the NO-sensitive soluble guanylyl cyclase, sGC) abolished the effect of STA on the relaxation of endothelium-intact rat thoracic arterial rings. In contrast, neither indomethacin (a non-selective COX inhibitor) nor 1400 W (an iNOS inhibitor) affect the STA effect (Fig. 2). These results indicated that protective effect of STA was dependent on NO signaling.

### STA increased NO, cGMP and BH4 production

**Fig. 3:**
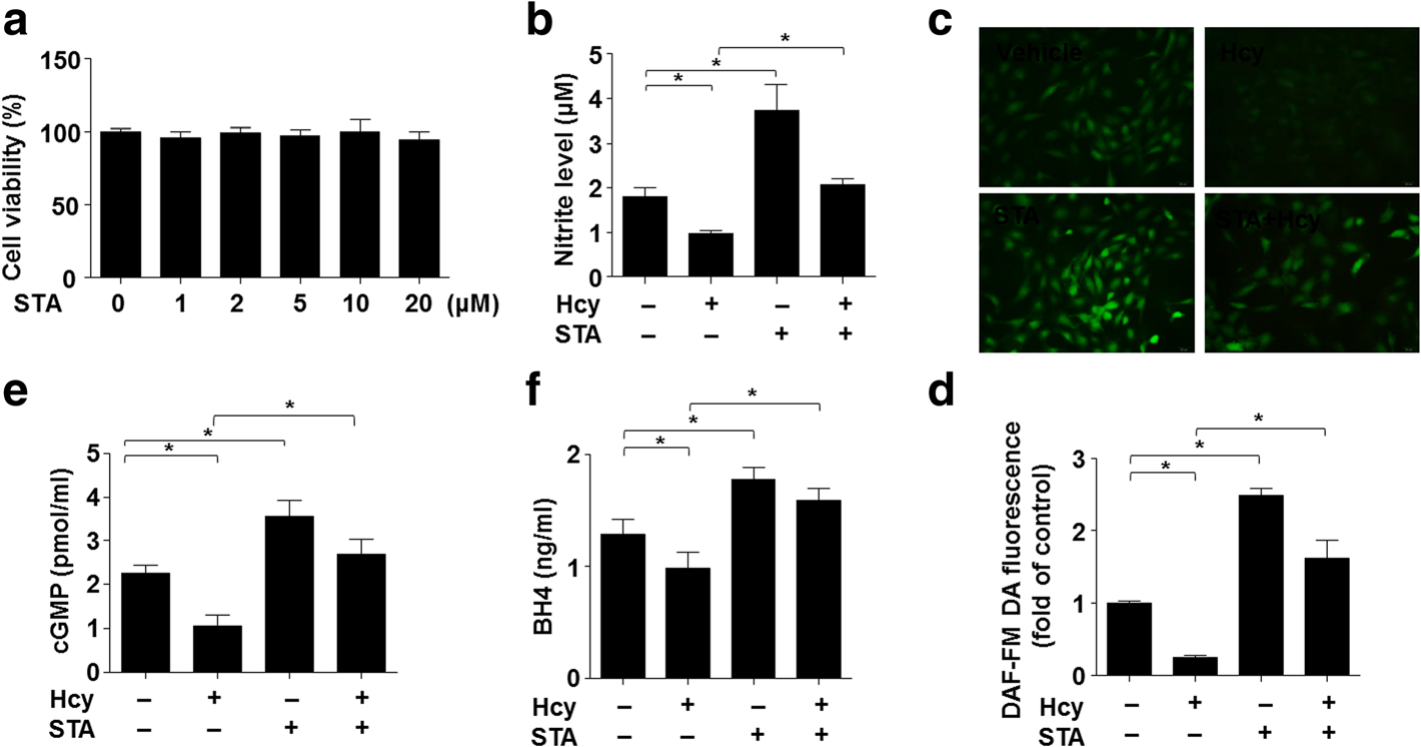
STA increased NO, cGMP and BH4 production. **a** BAECs were treated with indicated concentrations of STA for 24 h, and cell viability was measured by using MTT. **b** BAECs were pre-incubated with STA (10 μM) or vehicle for 12 h, and then exposed to Hcy (500 μM, 12 h). NO concentration in supernatants was measured by using the Griess reagent. **c** Intracellular NO was detected by using DAF-FM probe (40 × objective). **d** The mean fluorescence intensity was evaluated as in (**c**). **e** Rat thoracic artery rings pre-incubated with STA or vehicle were exposed to Hcy. The cGMP production was measured by using an enzyme immunoassay kit. **f** Intracellular levels of BH4 were measured by using BH4 ELISA Kit. Data shown were mean ± SEM, *n* = 5, ^*^P < 0.05

Thus, we examined the effect of STA on NO production in ECs. In cultured BAECs, treatment with STA (0–20 μM for 24 h) had no significant effect on cell viability, as measured with the MTT assay (Fig. 3a). The production of NO was significantly reduced in BAECs after the exposure to Hcy (500 μM for 12 h). However, pretreatment with STA (10 μM, 12 h) effectively attenuated the detrimental effect of Hcy on NO production (Fig. 3b). The effect of STA on NO production was confirmed with laser confocal microscopy using a specific fluorescence dye DAF-FM (Fig. 3c and d). Since endothelium-derived NO acts on arterial walls to promotes vasorelaxation by producing cGMP via the activation of sGC (Papapetropoulos et al., 2015), we further determined the cGMP levels in arterial rings treated with STA. As shown in Fig. 3e, The Hcy-decreased cGMP production was prevented by STA. BH4 is an essential cofactor for eNOS to completely couple NADPH oxidation to NO production. As shown in Fig. 3f, treatment with Hcy (500 μM, 12 h) significantly reduced BH4 production compared with vehicle control. However, pretreatment with STA effectively restored the BH4 level in BAECs. These results suggest that increase of BH4 and the ensuing activation of NO pathway may contribute to the protective effect of STA.

### STA ameliorated Hcy-induced eNOS uncoupling

**Fig. 4:**
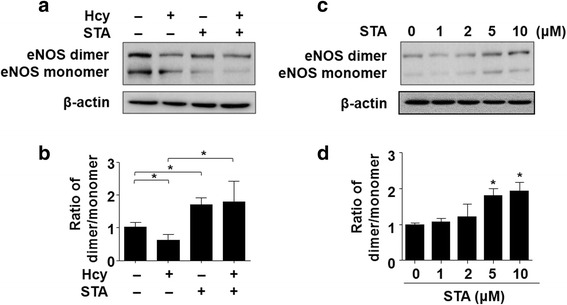
STA ameliorated Hcy-induced eNOS uncoupling. **a** BAECs were pre-incubated with STA (10 μM, 12 h) or vehicle and, then exposed to Hcy (500 μM, 12 h). Protein levels of eNOS dimers and monomers were detected by using low-temperature SDS-PAGE/western blotting. **c** BAECs were exposed to indicated concentrations of STA for 24 h, cell lysates were analyzed to determine eNOS dimers and monomers protein levels. **b** and **d** Quantification of eNOS dimer/monomer levels. Data were shown as mean ± SEM, *n* = 5. ^*^P < 0.05

It is established that BH4 plays an important role in facilitating eNOS dimerization, which is necessary for its normal catalytic function. Thus, we investigated the effects of STA on eNOS dimerization. As shown in Fig. 4a and b, the ratio of eNOS dimers to monomers, assayed by low-temperature SDS-PAGE, was significantly decreased in Hcy-treated ECs. However, pretreatment with STA ameliorated Hcy-decreased ratio of eNOS dimer to monomer. In fact, we observed that STA increased the dimer/monomer ratio in a dose-dependent manner (Fig. 4c and d). These results suggest that STA attenuated Hcy-induced eNOS uncoupling.

### STA increased the levels of GTPCH1 and DHFR

**Fig. 5:**
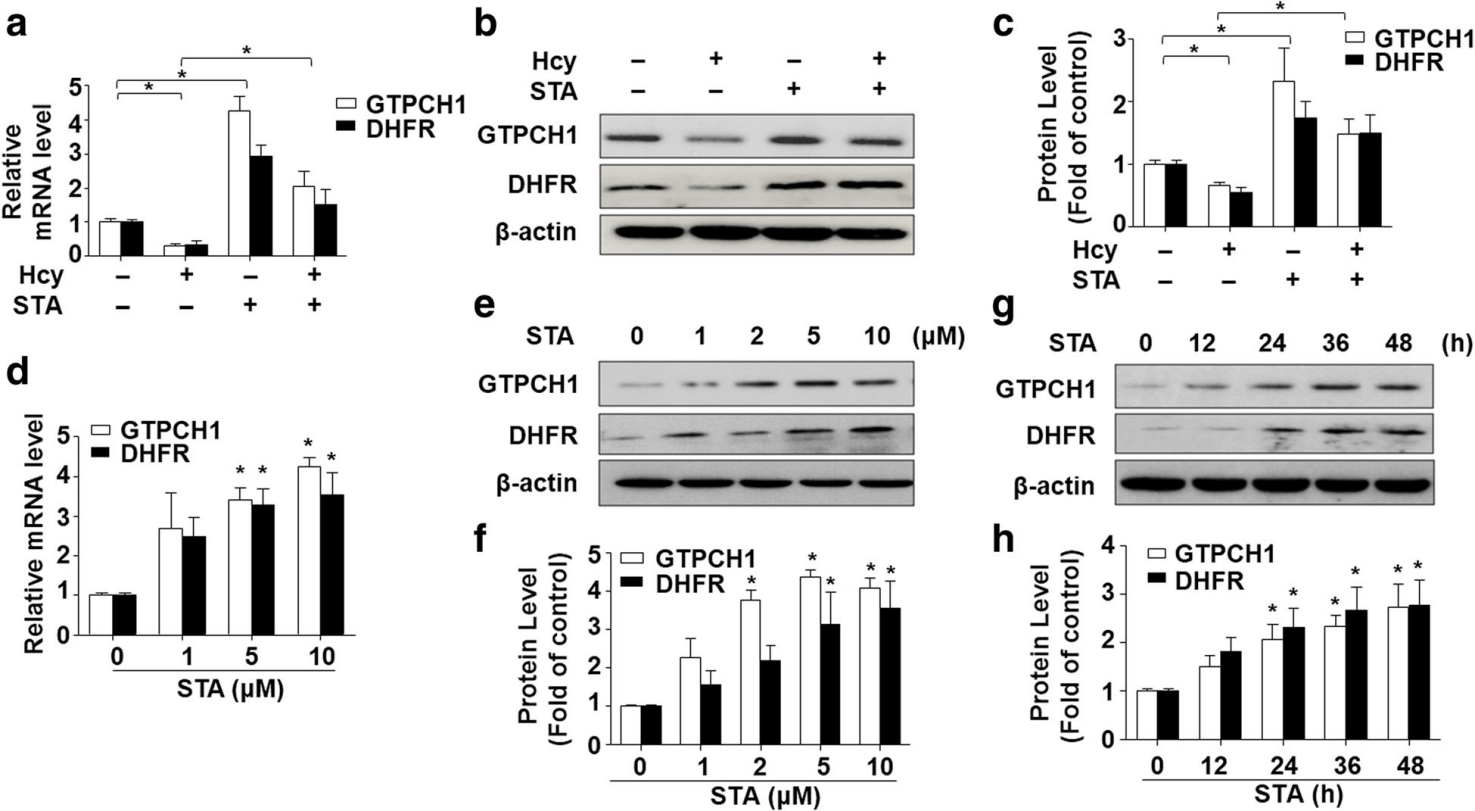
STA increased the levels of GTPCH1 and DHFR. **a** Relative mRNA levels of GTPCH1 and DHFR were assessed by using qRT-PCR. **b** Protein levels were assessed by using Western blotting. **c** Quantification of GTPCH1 and DHFR protein levels, which was normalized to the β-actin levels. **d** The mRNA levels of GTPCH1 and DHFR were assessed by using qRT-PCR. **e** GTPCH1 and DHFR protein levels were assessed by using Western blotting. **f** Quantification of GTPCH1 and DHFR protein levels as in (**e**). **g** BAECs were incubated with 10 μM STA for indicated time periods, GTPCH1 and DHFR protein levels were assessed by using Western blotting. **h** Quantification of GTPCH1 and DHFR protein levels as in (**g**). Data shown were mean ± SEM, *n* = 5. ^*^P < 0.05

GTPCH1 and DHFR are the two key catalyzing the biosynthesis of BH4 and NO. Therefore, we further assessed the effect of STA on the expression of these two genes in ECs by using qRT-PCR and Western blotting. As shown in Fig. 5a-c, Hcy reduced the mRNA and protein levels of GTPCH1 and DHFR, which were significantly reversed by STA pretreatment. Furthermore, STA increased mRNA and protein levels of GTPCH1 and DHFR in dose- and time-dependent manners (Fig. 5d-h). These results suggested that induction of GTPCH1 and DHFR may contribute to the protection against the Hcy-impaired BH4 and NO production.

### STA increased the expressions of GTPCH1 and DHFR via activation of Nrf2

**Fig. 6:**
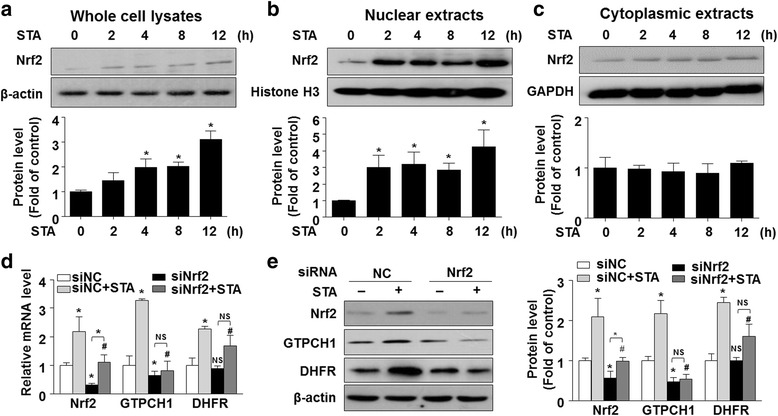
STA increased the expressions of GTPCH1 and DHFR via Nrf2 activation. Western blotting analysis of Nrf2 protein levels in whole (**a**), nuclear (**b**) and cytoplasmic (**c**) fractions. Quantification of Nrf2 protein levels as in (**a**, **b** and **c** (bottom)). β-actin, Histone H3 and GAPDH were used for internal controls in whole, nuclear and cytoplasmic fractions, respectively. **d** ECs were transfected with scrambled siRNA or Nrf2 siRNA for 24 h and then treated with STA (10 μM) for another 24 h. The mRNA levels of Nrf2, GTPCH1 and DHFR were assessed by using qRT-PCR. **e** Western blotting analysis of Nrf2, GTPCH1 and DHFR protein levels. Quantification of Nrf2, GTPCH1 and DHFR protein levels as in H (right, *n* = 3). Data shown were mean ± SEM. *P < 0.05

Nuclear factor erythroid 2–related factor 2 (Nrf2) transcriptionally controls the gene expression of many cytoprotective enzymes, such as heme oxygenase-1 (HO-1) (Swamy et al., 2016) and NAD(P)H:quinone reductase (NQO1) (Rohrer et al., 2014). Particularly, it was shown that Nrf2 regulated-GTPCH1/BH4 axis ameliorated skin injury (Xue et al., 2017). To test whether Nrf2 is involved in the effects of STA on the induction of expressions of GTPCH1 and DHFR, BAECs were treated with STA (10 μM) for different time periods and the Nrf2 levels were detected by using western blotting. As shown in Fig. 6a, STA increased the total Nrf2 protein level. Furthermore, STA treatment rapidly increased Nrf2 protein level in the nuclear portions (Fig. 6b and c), suggesting that Nrf2 is activated and likely translocated into the nuclear compartment in response to STA exposure. Furthermore, Nrf2 knockdown by siRNA abrogated STA-increased levels of GTPCH1 and DHFR (Fig. 6d and e). These results indicated that activation of Nrf2 might act as a transcriptional regulator to mediate the STA-induced gene expression of GTPCH1 and DHFR.

## Discussion

Endothelial dysfunction has been implicated in the pathogenesis of CVDs such as atherosclerosis, diabetes and hypertension (Martin-Timon et al., 2014). STA is belongs to naturally occurring alkaloids. Among their diverse pharmacological properties such as anti-microbial, anti-tumor, anti-diabetic and anti-inflammatory effects, many natural alkaloids have been reported to possess vascular protective activities (Kittakoop et al., 2014; Cushnie et al., 2014). However, the molecular mechanisms underlying such vascular benefit remain largely unknown. In the present study, we for the first time provided both in vitro and ex vivo evidence that STA protects against the Hcy-impaired endothelial dysfunction. Most importantly, we defined the novel mechanisms by which STA enhances eNOS coupling and NO bioavailability via up-regulation of GTPCH1 and DHFR, the genes for two major enzymes responsible for the de novo biosynthesis and salvage pathways of BH4. Given the critical roles of NO signaling in the vascular homeostasis and diseases, these findings are of pathophysiological importance to our understanding of the pharmacological mechanisms by which the naturally occurring compounds exert their vascular actions.

Hcy is an intermediate metabolite in the metabolic pathway of cysteine and methionine (Gurda et al., 2015). Elevated level of plasma Hcy is an independent risk factor of CVD and Hcy contributes to the development of CVD as it causing endothelial dysfunction (Ivanov et al., 2015). In our study, we found that STA prevented Hcy-induced impairment of endothelium-dependent relaxation (Fig. 1). L-NAME, a NOS inhibitor, suppressed the relaxation of STA, indicating that the vasorelaxant effect of STA was associated with NO release. This notion is corroborated with the result that ODQ, a NO-sensitive guanylyl cyclase inhibitor that blocks the signaling pathway of the endothelium-derived NO, also reduced STA-induced relaxation. By contrast, iNOS did not play any roles in the enhanced NO release, which was confirmed using the iNOS inhibitor 1400 W (Fig. 2). These results clearly demonstrated that STA can elicit a NO-dependent vasodilation.

ECs produce a variety of vasoactive substances, such as NO, endothelium-derived hyperpolarizing factor (EDHF), prostacyclin, or endothelin-1 (ET-1), Ang II, thromboxane A2 and prostaglandin H2, to keep the delicate balance between vasodilation and vasoconstriction (Husain et al., 2015). Among these endothelial-derived vasodilators, NO is the most important one because of its key role in inhibiting platelet aggregation, inflammation, oxidative stress, vascular smooth muscle cell migration and proliferation, and leukocyte adhesion (Zhao et al., 2015). In our study, we showed that the impaired production of NO by Hcy was rescued by STA (Fig. 3). NO-induced relaxation is associated with increased levels of cGMP in vascular smooth muscle cells. An elevation in intracellular cyclic GMP levels leads to vasorelaxation. Our findings indicated that the effect of STA-induced relaxation via NO-dependent cGMP production. eNOS catalyzes the biosynthesis of NO in endothelial cells whereas BH4 is a critical determinant of eNOS activity. We demonstrated that STA prevented the Hcy-induced BH4 decrease and that STA increased BH4 production in ECs (Fig. 3). BH4 bioavailability in the vasculature appears to be regulated by both a de novo pathway using the rate-limiting enzyme GTP-cyclohydrolase I (GTPCH1) and a salvage pathway from the synthetic pterin, sepiapterin, which is metabolized to BH4 by sepiapterin reductase (SR) and endothelial dihydrofolate reductase (DHFR) (Bendall et al., 2014). It has been previously demonstrated that depletion of BH4 renders uncoupling eNOS uncoupled from L-arginine oxidation, resulting in generation of O2^−^ rather than NO. Such an “eNOS uncoupling” status is thought to contribute to vascular oxidative stress and endothelial dysfunction (Hsieh et al., 2014). In fact, it was previously described that homocysteine exposure could lead to eNOS uncoupling in EC (Topal et al., 2004). The mechanisms underlying the suppressive effects of NO bioavailability and eNOS uncoupling are still incompletely understood. In light of the previous findings that Hcy significantly reduced intracellular BH4 level in ECs and that ascorbic acid, a BH4 regenerator, rescued the NO production in Hcy-exposed ECs (Topal et al., 2004), a decreased in BH4 level or, more importantly, the BH4:BH2 ratio (Crabtree et al., 2009), may account for the deleterious effect of homocysteine. Interestingly, we found STA markedly increased eNOS coupling, as suggested by the increased levels of NO production, dimerized eNOS, BH4:BH2 ratio, as well as GTPCH1 and DHFR expression in ECs (Figs. 4 and 5).

**Fig. 7:**
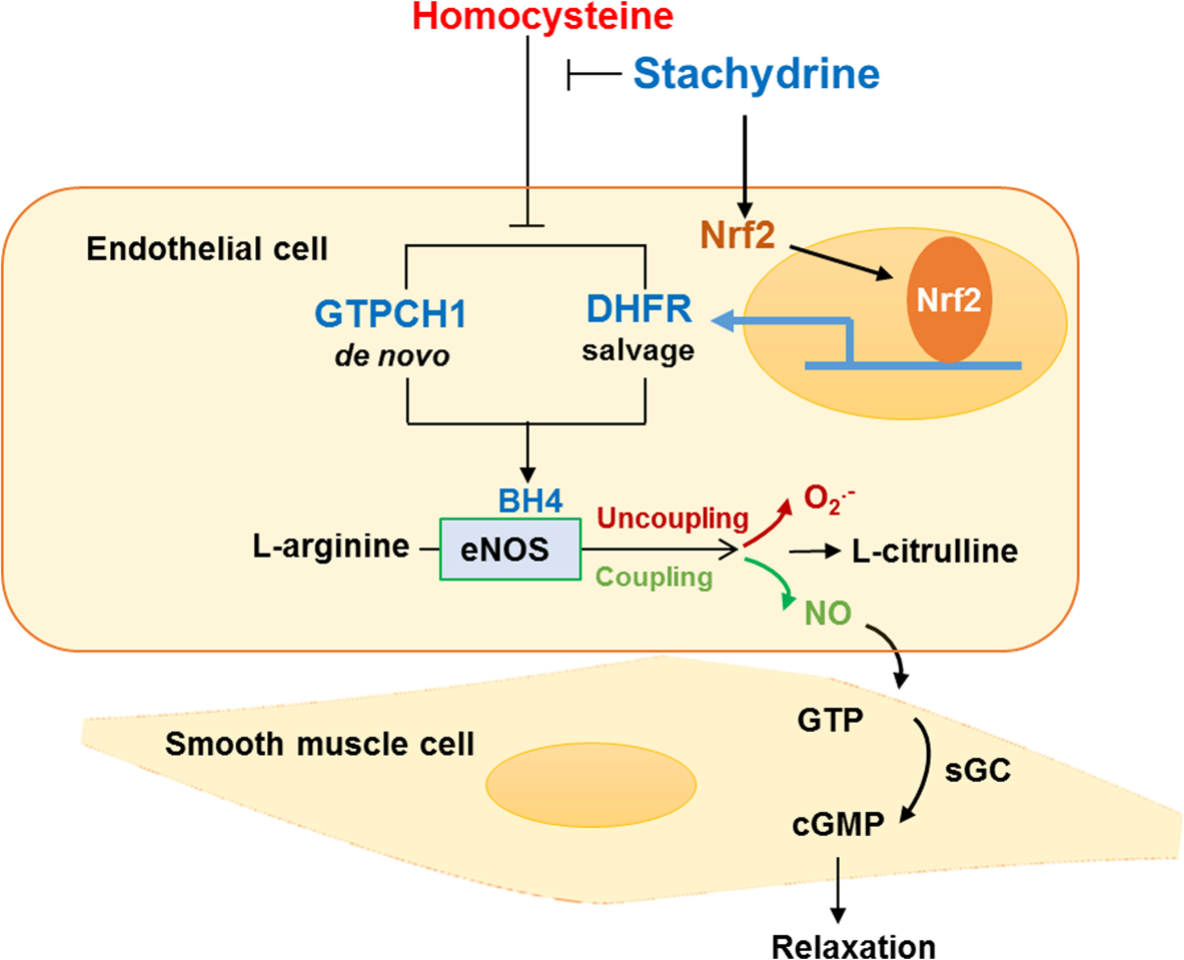
Schematic model of the effect of STA on Hcy-induced endothelial dysfunction. Stachydrine ameliorated Hcy-induced endothelial dysfunction via Nrf2-dependent up-regulation of GTPCH1 and DHFR. The de novo synthesis and salvage of BH4 reversed eNOS uncoupling and increased NO production, thus, leading to improved endothelium-dependent vasorelaxation

Nrf2 is sequestered in the cytoplasmic portion and bind to its repressor molecule, Keap1 (Soares et al., 2016). Under stressful conditions such as oxidative or ER stress, Nrf2 dissociates from the Nrf2-Keap1 complex. Subsequently, Nrf2 undergoes nuclear translocation to transcriptionally activate its target genes. In the present study, Nrf2 was rapidly increased by STA in ECs. Nrf2 was increased in the nuclear portion within 2 h after the STA treatment, indicating that Nrf2 activation is an early event in the STA signaling. A critical role of Nrf2 was further supported by the results that gene silencing of Nrf2 attenuated the STA induction of both GTPCH1 and DHFR (Fig. 6). Given that both GTPCH1 and DHFR genes harbor the cognate cis-elements within the 5′-flanking regions, we speculate that STA may activate Nrf2 to transcriptionally up-regulate the gene expression of GTPCH1 and DHFR, which are responsible for the de novo biosynthesis and salvage pathways of BH4; such a coordinated gene induction result in increased BH4 and NO bioavailability, thus protecting endothelial-dependent vascular function against such metabolic risk factor as homocysteinemia, saturated free fatty acids and Ang II (Fig. 7).

## Conclusion

Taken together, our results demonstrated that stachydrine ameliorated Hcy-induced endothelial dysfunction via Nrf2 dependent up-regulation of GTPCH1 and DHFR and increase in bioavailabilities of BH4 and NO. These results revealed a new mechanism by which the natural alkaloids STA protect endothelial function in response to such risk factors as increased concentration of homocysteine.

## Additional files

Additional file 1: **Figure S1.** STA slightly improved the vasorelaxation in rat TA, MA, and RA. (TIFF 289 kb)

Additional file 2: **Figure S2.** STA improved Ang II or PA-impaired vascular relaxation. (TIFF 258 kb)

## Abbreviations

BH4: Tetrahydrobiopterin
CVDs: Cardiovascular diseases
DHFR: Dihydrofolate reductase
ECs: Endothelial cells
eNOS: Endothelial nitric oxide synthase
GTPCH1: Guanosine triphosphate cyclohydrolase-1
Hcy: Homocysteine
L-NAME: NG-nitro-L-arginine methylester
MA: Mesenteric arteries
NO: Nitric oxide
Nrf2: Nuclear factor erythroid 2–related factor 2
ODQ: 1H- [1, 2, 4] Oxadiazolo [4,3-a] quinoxalin-1-one
RA: Renal arteries
STA: Stachydrine
TA: Thoracic aortas
