# Supplementary figures and images for: Stachydrine protects eNOS uncoupling and ameliorates endothelial dysfunction induced by homocysteine
Source: Mol Med. 2018 Mar 19;24:10. doi: 10.1186/s10020-018-0010-0 (PMC6016886; doi:10.1186/s10020-018-0010-0)

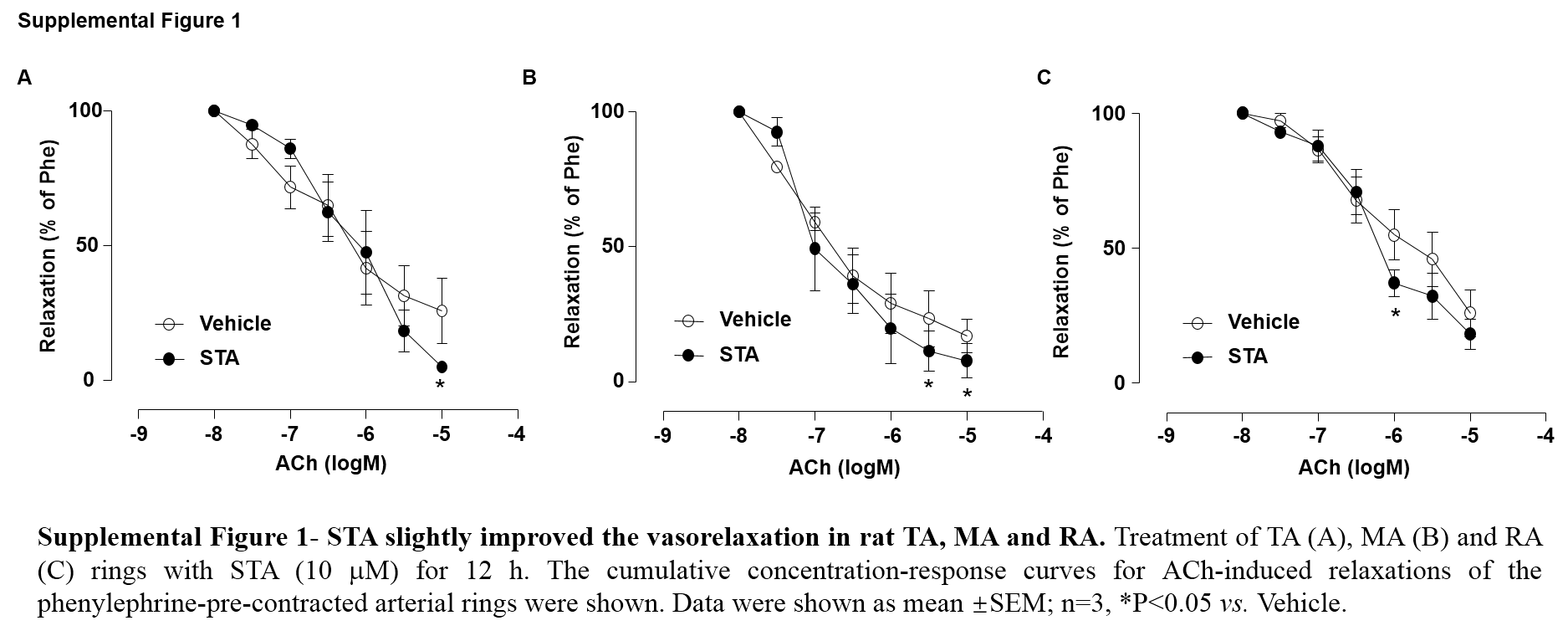

Supplement: Supplementary file 1 — Figure S1. STA slightly improved the vasorelaxation in rat TA, MA, and RA. (TIFF 289 kb) [file 10020_2018_10_MOESM1_ESM.tif]

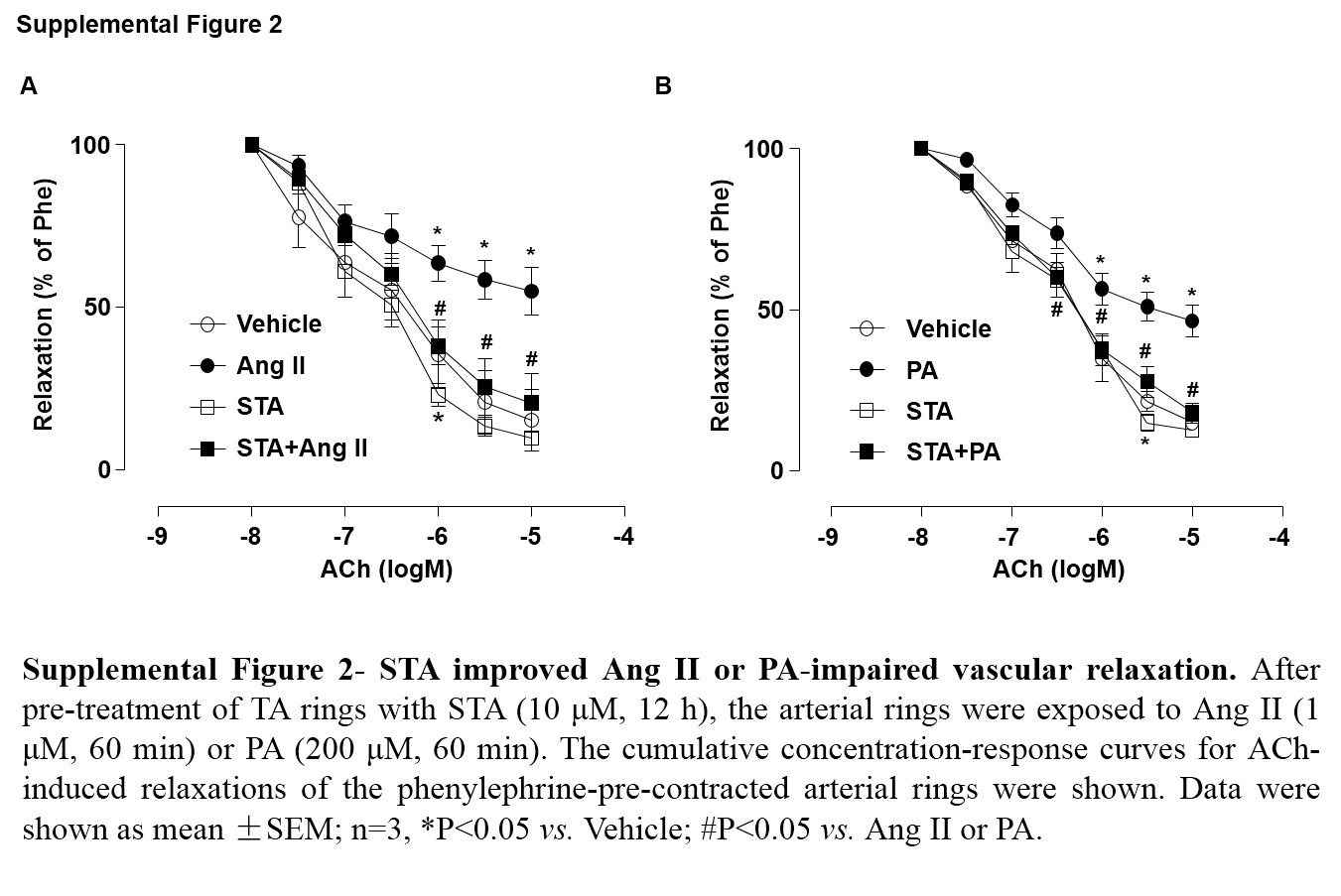

Supplement: Supplementary file 2 — Figure S2. STA improved Ang II or PA-impaired vascular relaxation. (TIFF 258 kb) [file 10020_2018_10_MOESM2_ESM.tif]
